# From fields to eyes: the epidemiology and immune challenges of fungal keratitis

**DOI:** 10.3389/fcimb.2026.1768611

**Published:** 2026-04-02

**Authors:** Yu Li, Xiao-lu Zhang, Bao-hua Huang, Qi Zhao, Yu-feng Gu

**Affiliations:** Department of Clinical Laboratory Center, Yantai Yuhuangding Hospital, Yantai, Shandong, China

**Keywords:** cytokines, epidemiology, fungal keratitis, immunosuppression, microenvironment

## Abstract

**Objectives:**

To investigate the regional epidemiological characteristics, immune mechanisms, and potential therapeutic strategies for fungal keratitis (FK) in order to provide evidence for improving patient outcomes.

**Methods:**

Clinical data from 145 patients diagnosed with FK between 2018 and 2024 were collected to analyze epidemiological features and predisposing factors. Aqueous humor (AqH) and peripheral blood samples from a subset of patients underwent flow cytometric analysis and cytokine profiling, with glaucoma patients serving as controls. The composition, function, and cytokine profiles of immune cells in the AqH of FK patients were characterized to elucidate the ocular immune microenvironment.

**Results:**

The majority of FK patients were middle-aged and elderly males (male-to-female ratio: 1.9:1), with agricultural injuries representing the predominant etiology (73.10%) and Fusarium species identified as the leading pathogen (44.12%). Peak incidence occurred in October and November, coinciding with local harvest activities. In the AqH, neutrophils were the predominant infiltrating cells (81.25%), accompanied by a significantly elevated proportion of B cells (30.70%) and marked reductions in both the proportion and function of T cells and NK cells. Concentrations of IL-6 and IL-8 in the AqH were approximately 20-fold and 200-fold higher, respectively, than in controls. Immunosuppressive cell populations, including regulatory B (Breg) cells, activated regulatory T (Treg) cells, and polymorphonuclear myeloid-derived suppressor cells (PMN-MDSCs), were significantly expanded. Additionally, hyperglycemic patients exhibited heightened inflammatory responses, with significantly elevated concentrations of pro-inflammatory cytokines (IL-4, IL-6, IL-8, and TNF-α) in their AqH compared with normoglycemic patients.

**Conclusions:**

FK in this region is strongly associated with agricultural activities. Patients demonstrated diminished adaptive immune function alongside expansion of immunosuppressive cell populations, resulting in an ocular microenvironment characterized by suppressed adaptive immunity coexisting with enhanced innate immune responses. IL-6 may play a central role in shaping this immunosuppressive microenvironment by amplifying pro-inflammatory responses and inhibiting T cell function. Hyperglycemia appears to further exacerbate immune dysregulation, potentially impacting prognosis. Early therapeutic targeting of IL-6 may represent a strategy to alleviate ocular inflammation and reduce complications in FK; however, this hypothesis requires validation in future studies.

## 1 Introduction

Fungal keratitis (FK) is a severe corneal infection caused by fungal invasion of corneal tissue and represents a major cause of infectious ocular disease worldwide (Raj et al., 2021). Among the approximately 105 known pathogenic fungi, FK is most commonly caused by *Aspergillus*, *Fusarium*, and *Candida* species, which collectively account for approximately 70% of all cases. FK caused by *Rhizopus*, *Mucor*, and other fungi is relatively rare (Charlene H et al., 2025). Fungal infection can lead to the formation of corneal ulcers, potentially resulting in tissue necrosis, scarring, and, in severe cases, blindness. A 2001 World Health Organization survey indicated that corneal blindness is the second leading cause of blindness globally, after cataracts, with FK representing a key contributing factor, particularly in developing countries.

Trauma, corneal abrasions, and contact lens use are the principal risk factors for FK. Patients typically present with an insidious onset and gradual progression, with characteristic clinical manifestations including severe ocular pain, conjunctival hyperemia, photophobia, blurred vision, and a pronounced foreign body sensation. Slit-lamp examination frequently reveals hypopyon in the anterior chamber, and the presence of satellite lesions surrounding the ulcer is a hallmark feature of FK. Standard diagnostic modalities include comprehensive ophthalmic examination, *in vivo* confocal microscopy, corneal scraping with microscopy, and fungal culture. Although early diagnosis and standardized treatment can effectively preserve vision in FK patients, in some cases, even aggressive therapy fails to control the spread of infection or preserve visual function. For such patients, surgical intervention or corneal transplantation is often required (Rameshkumar et al., 2023). Recent epidemiological studies continue to underscore the global burden of FK, with considerable regional variation in prevalence and causative pathogens (Brown et al., 2021; , Durand et al., 2023).

In contrast to bacterial keratitis, where acute inflammation often facilitates rapid pathogen clearance, FK frequently follows a more chronic and indolent course, suggesting distinct mechanisms of immune-mediated tissue damage. The host immune response in FK is complex, involving both innate and adaptive components, and an imbalanced or dysregulated response may contribute to persistent inflammation and corneal scarring rather than effective pathogen eradication. Immune cells and cytokines may paradoxically contribute to corneal damage during the process of combating infection (Zhang and He, 2024). Neutrophils, serving as the primary defense against fungal pathogens, are rapidly recruited to the site of infection in the early stages, directly participating in pathogen clearance through phagocytosis, release of lysosomal enzymes, and secretion of antimicrobial peptides. However, excessive neutrophil activation and accumulation may exert deleterious effects. For instance, the release of elastase and gelatinase can limit the spread of infection by altering tissue architecture, yet these enzymes may also compromise normal tissue function. The generation of large quantities of reactive oxygen species (ROS) can induce lipid peroxidation of cell membranes, thereby damaging the integrity of corneal epithelial cells (Sharma et al., 2022). Neutrophils also release various pro-inflammatory cytokines that recruit additional immune cells, including macrophages and lymphocytes, to the site of infection. Once activated, these cells produce cytokines such as IL-1β, TNF-α, IL-6, and IL-8, which, while contributing to pathogen killing, can also damage the corneal extracellular matrix, impairing corneal transparency and ultimately leading to visual impairment (Rai et al., 2021).

Therefore, a comprehensive understanding of the immune characteristics of the ocular microenvironment in FK patients is not only essential for elucidating the immunological mechanisms underlying the onset and progression of ocular inflammation but also carries significant clinical implications for the targeted modulation of specific cytokines to control inflammatory progression and improve patient prognosis.

## 2 Materials and methods

### 2.1 Data source

This study included 145 patients diagnosed with fungal keratitis (FK) at our institution between January 2018 and January 2024. Detailed clinical data were collected, including occupation, trauma history, prior ophthalmic surgical history, and contact lens use.

Routine aqueous humor (AqH) sampling is not indicated in patients with FK. In this study, AqH specimens were obtained exclusively from FK patients who underwent anterior chamber paracentesis for severe disease. Anterior chamber paracentesis in the context of FK is warranted only under specific clinical circumstances: high clinical suspicion for endophthalmitis, severe anterior chamber inflammation with persistent hypopyon, or diagnostic uncertainty necessitating differentiation between infectious and non-infectious etiologies. Patients were excluded if they had concurrent ocular infections, a history of autoimmune disease, or were receiving systemic immunosuppressive therapy. AqH samples were collected following the initiation of antifungal therapy, reflecting either advanced disease or failure to respond to initial treatment. Of the FK patients who underwent anterior chamber paracentesis, 12 yielded sufficient cellular material for immunological analysis.

The control group comprised 12 patients undergoing surgical intervention for glaucoma during the same period, selected to provide comparable intraocular fluid samples. We acknowledge that glaucoma may be associated with low-grade intraocular inflammation, which could potentially confound baseline cytokine levels. However, given the significant ethical and practical constraints inherent in obtaining AqH samples from healthy individuals, the use of glaucoma patients as a control group represents a methodologically reasonable and clinically justifiable approach.

### 2.2 Diagnosis of FK

Following instillation of topical anesthetic eye drops, corneal scrapings were obtained directly from the ulcer bed and margins under slit-lamp biomicroscopy. Specimens were examined microscopically after treatment with 10% potassium hydroxide (KOH) and Gram staining. Concurrently, corneal scrapings or tissue biopsy specimens were inoculated onto Sabouraud dextrose agar and blood agar. All cultures were incubated at both 37 °C and 25 °C for a minimum of two weeks, and positive cultures were subsequently identified.

In selected patients, *in vivo* confocal microscopy (IVCM) was performed using the Heidelberg Retina Tomograph III with Rostock Cornea Module (HRTIII-RCM; Heidelberg Engineering GmbH, Dossenheim, Germany). The morphology, distribution, and depth of fungal elements within the cornea were evaluated at 800× magnification.

A diagnosis of FK was established, and antifungal therapy was initiated when either IVCM or corneal scraping microscopy yielded positive findings.

### 2.3 Handling of aqueous humor and peripheral blood samples

To investigate the immune status of the aqueous humor (AqH) in FK patients, AqH specimens were collected from patients undergoing anterior chamber paracentesis. Samples were obtained from patients requiring surgical intervention (e.g., anterior chamber irrigation or corneal transplantation) following initiation of antifungal therapy; these samples therefore reflect either advanced disease or inadequate response to initial medical treatment. Under surgical microscopy, approximately 100 μL of AqH was aspirated from each eye, followed by anterior chamber irrigation. The irrigation fluid was collected in sterile tubes, centrifuged at 400 × g for 8 minutes, and the resulting cell pellet was immediately processed for flow cytometric analysis.

Concurrently, two EDTA-anticoagulated peripheral venous blood samples were collected from each patient. One sample was used directly for flow cytometric analysis, while the other was centrifuged at 2500 × g for 10 minutes, and the plasma supernatant was aliquoted and stored at −80 °C for subsequent cytokine analysis.

### 2.4 Flow cytometric analysis

Appropriate volumes of aqueous humor or EDTA-anticoagulated peripheral blood specimens were incubated with the corresponding antibody panels in the dark at room temperature for 20 minutes. Following incubation, 2 mL of erythrocyte lysis buffer was added, and samples were centrifuged at 500 × g for 5 minutes; the supernatant was then discarded. Cells were washed once with 2 mL phosphate-buffered saline (PBS), centrifuged at 500 × g for 5 minutes, and the supernatant was discarded. The cell pellet was resuspended in 500 μL PBS for flow cytometric analysis.

Data acquisition was performed using a BD FACSCanto II flow cytometer with FACSDiva software (version 7.1; BD Biosciences, San Jose, CA, USA). The following antibodies were purchased from BD Biosciences (San Jose, CA, USA): anti-CD3, anti-CD4, anti-CD8, anti-CD19, anti-CD45, anti-CD25, anti-CD127, anti-CD28, anti-CD45RA, anti-CD16, anti-CD33, anti-CD14, and anti-CD15. Anti-CD138, anti-CD24, anti-CD27, anti-CD20, anti-CD38, and anti-IFN-γ antibodies were purchased from BioLegend (San Diego, CA, USA).

Data analysis was performed using Kaluza software (Beckman Coulter, Brea, CA, USA). The gating strategy employed CD45 expression in conjunction with forward scatter (FSC) and side scatter (SSC) characteristics to identify leukocyte populations, including granulocytes, monocytes, and lymphocytes. Lymphocyte subsets and functional characteristics were further delineated by analyzing the expression of additional cluster of differentiation markers.

**Figure 1:**
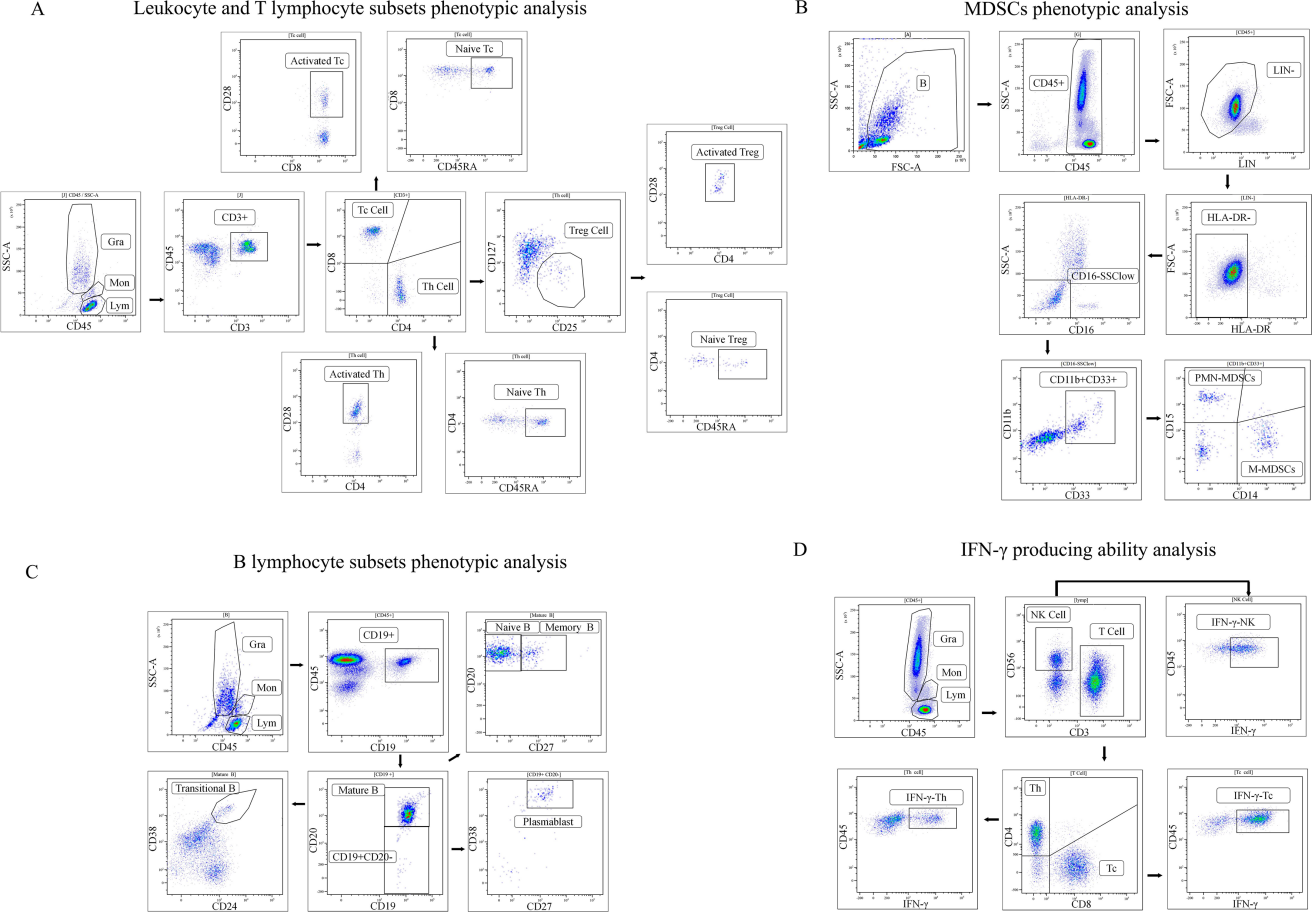
Flow cytometric gating strategy for leukocyte identification, immune cell subset characterization, and functional analysis. **(A)** Gating strategy for leukocyte and lymphocyte subset identification. **(B)** Gating strategy for myeloid-derived suppressor cell (MDSC) phenotype analysis. **(C)** Gating strategy for B cell and B cell subset identification. **(D)** Gating strategy for assessment of IFN-γ production capacity in T cells and NK cells.

For T cell analysis, CD3^+^T cells were identified, and CD4^+^helper T (Th) cells and CD8^+^ cytotoxic T (Tc) cells were subsequently gated based on CD3, CD4, and CD8 expression. Within the Th cell population, regulatory T cells (Treg) were defined as CD25hiCD127low, and their functional status was assessed based on CD28 and CD45RA expression (**Figure 1A**).

For myeloid-derived suppressor cell (MDSC) analysis, the lineage-negative (LIN^−^) non-lymphocyte population was selected, and HLA-DR^−^cells were gated. After excluding granulocytes based on CD16 expression and SSC characteristics, CD11b^+^CD33^+^cells were identified. Monocytic MDSCs (M-MDSCs) were defined as CD11b^+^CD33^+^CD14^+^cells, and polymorphonuclear MDSCs (PMN-MDSCs) were defined as CD11b^+^CD33^+^CD15^+^ cells (**Figure 1B**).

For B cell subset analysis, CD19^+^B cells were identified within the lymphocyte gate. B cell differentiation stages were classified based on the expression of CD138, CD24, CD27, CD20, and CD38 (**Figure 1C**).

To evaluate the IFN-γ production capacity of NK cells and T cells, lymphocytes were stimulated with phorbol 12-myristate 13-acetate (PMA) and ionomycin. Intracellular IFN-γ was retained using a protein transport inhibitor, and intracellular IFN-γ expression levels in Th cells, Tc cells, and NK cells were subsequently analyzed using anti-IFN-γ antibodies (**Figure 1D**).

### 2.5 Cytokine analysis

Cytokine concentrations in aqueous humor and plasma samples were measured using the Cytometric Bead Array (CBA) Human Inflammatory Cytokine Kit (BD Biosciences, San Jose, CA, USA), which enables simultaneous quantification of 12 cytokines: IL-1β, IL-2, IL-4, IL-5, IL-6, IL-8, IL-10, IL-12p70, IL-17A, IFN-α, IFN-γ, and TNF-α. Briefly, 25 μL of each sample was incubated with 25 μL of capture antibody-coated beads and 25 μL of phycoerythrin (PE)-conjugated detection antibodies in the dark at room temperature for 3 hours. Following incubation, 1 mL of wash buffer was added to each tube, and samples were centrifuged at 200 × g for 5 minutes. The supernatant was discarded, and the bead pellet was resuspended in 100 μL of wash buffer for acquisition on the flow cytometer. Data were analyzed using FCAP Array software (BD Biosciences). Cytokine assays were performed on aqueous humor and peripheral blood samples from 12 FK patients and 12 control subjects.

### 2.6 Statistical analysis

Statistical analyses were performed using GraphPad Prism software (version 9.0; GraphPad Software, San Diego, CA, USA). Demographic and clinical data were summarized using descriptive statistics. For comparisons between two groups, the unpaired Student’s t-test was used when data were normally distributed or approximated normality; normality was assessed using the Shapiro-Wilk test. Although the aqueous humor sample size was limited (n=12 per group), the t-test remains appropriate for exploratory analysis when normality assumptions are satisfied or achieved through data transformation. For data that violated normality assumptions or for comparisons involving more than two groups, the nonparametric Kruskal–Wallis test was employed, followed by Dunn’s *post hoc* test for multiple comparisons. A two-tailed p-value < 0.05 was considered statistically significant.

## 3 Results

### 3.1 Predominance of middle-aged males with corneal trauma from agricultural injury as the primary etiological factor

Between January 2018 and January 2024, a total of 145 patients with FK were admitted to our institution (**Table 1**). The majority were middle-aged males engaged in agricultural occupations, with a male-to-female ratio of 1.9:1. Corneal trauma or foreign body injury constituted the primary etiological factors, although a subset of patients presented with ocular symptoms without a clearly identifiable antecedent cause. Cases of FK attributable to prior cataract surgery or contact lens wear were relatively uncommon. The interval between the onset of ocular symptoms or initial injury and first clinical presentation ranged from 1 day to 2 years, with a median time to presentation of 48 days.

**Table 1:** Basic information of 145 FK patients.

Feature	Details	n (%)
Sex	Male	96 (66.21)
	Female	49 (33.79)
	Male/Female	1.9:1
Age	Range	36-85
	30-40	2 (1.38)
	40-50	9 (6.21)
	50-60	41 (28.28)
	60-70	68 (46.90)
	70-80	19 (13.10)
	80-	6 (4.14)
	Median	64
Profession	Farmers	134 (92.41)
	Worker	10 (6.90)
	Retirees	1 (0.69)
Infection route	Corneal trauma	106 (73.10)
	Operation	3(73.10)
	Contact lenses	1 (0.69)
	Unknown	35 (24.14)

### 3.2 Seasonal peak of FK in October and November with *Fusarium* species as the predominant pathogen

**Figure 2:**
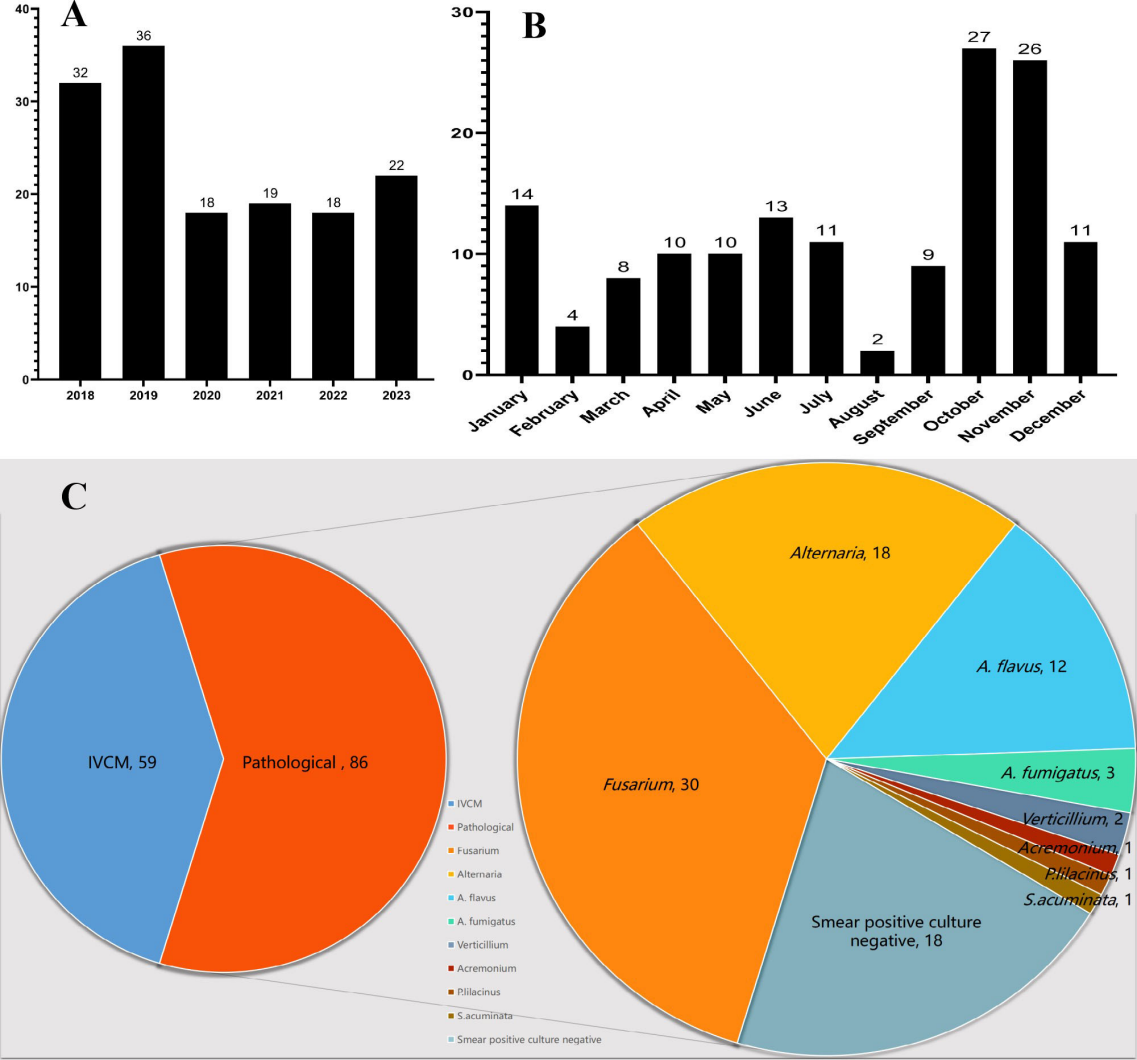
Temporal distribution and pathogen identification in 145 patients with fungal keratitis. **(A)** Annual distribution of FK cases. **(B)** Monthly distribution of FK cases. **(C)** Distribution of fungal pathogens isolated by culture.

A higher number of FK cases were diagnosed in 2018 and 2019 compared with subsequent years (**Figure 2A**), with October and November representing the peak months for FK incidence in this region (**Figure 2B**). Of the 145 cases included in this study, 59 (40.7%) were diagnosed by *in vivo* confocal microscopy (IVCM) based on the visualization of characteristic fungal hyphae, while the remaining 86 cases (59.3%) were diagnosed by corneal scraping with direct microscopic examination. Among the 86 patients with positive corneal scraping results, fungal pathogens were successfully isolated by culture in 68 cases (79.1%). The distribution of isolated pathogens was as follows: *Fusarium* spp. (n = 30; 44.1%), *Lichtheimia* spp. (n = 18; 26.5%), *Aspergillus flavus* (n = 12; 17.6%), *Aspergillus fumigatus* (n = 3; 4.4%), *Neoscytalidium* spp. (n = 2; 2.9%), and one case each (1.5%) of *Cladosporium* spp., *Penicillium aurantiogriseum*, and *Chaetomium* spp. (**Figure 2C**).

### 3.3 Neutrophil-dominant infiltration in aqueous humor of FK patients with B lymphocytes as the predominant lymphocyte subpopulation

Of the 18 FK patients who underwent anterior chamber irrigation, 12 yielded sufficient cellular material for immunological analysis. The control group comprised 12 glaucoma patients who underwent surgical intervention during the same period.

In the aqueous humor of FK patients, neutrophils were the predominant infiltrating cell population, accounting for approximately 81.25% of total nucleated cells—significantly higher than in the control group, consistent with an acute infectious process. Conversely, the proportion of lymphocytes was markedly reduced in FK patients (5.56%) compared with controls. No significant difference in monocyte proportions was observed between the two groups. In peripheral blood, no notable differences in the distribution of these three cell populations were detected between FK patients and controls.

**Figure 3:**
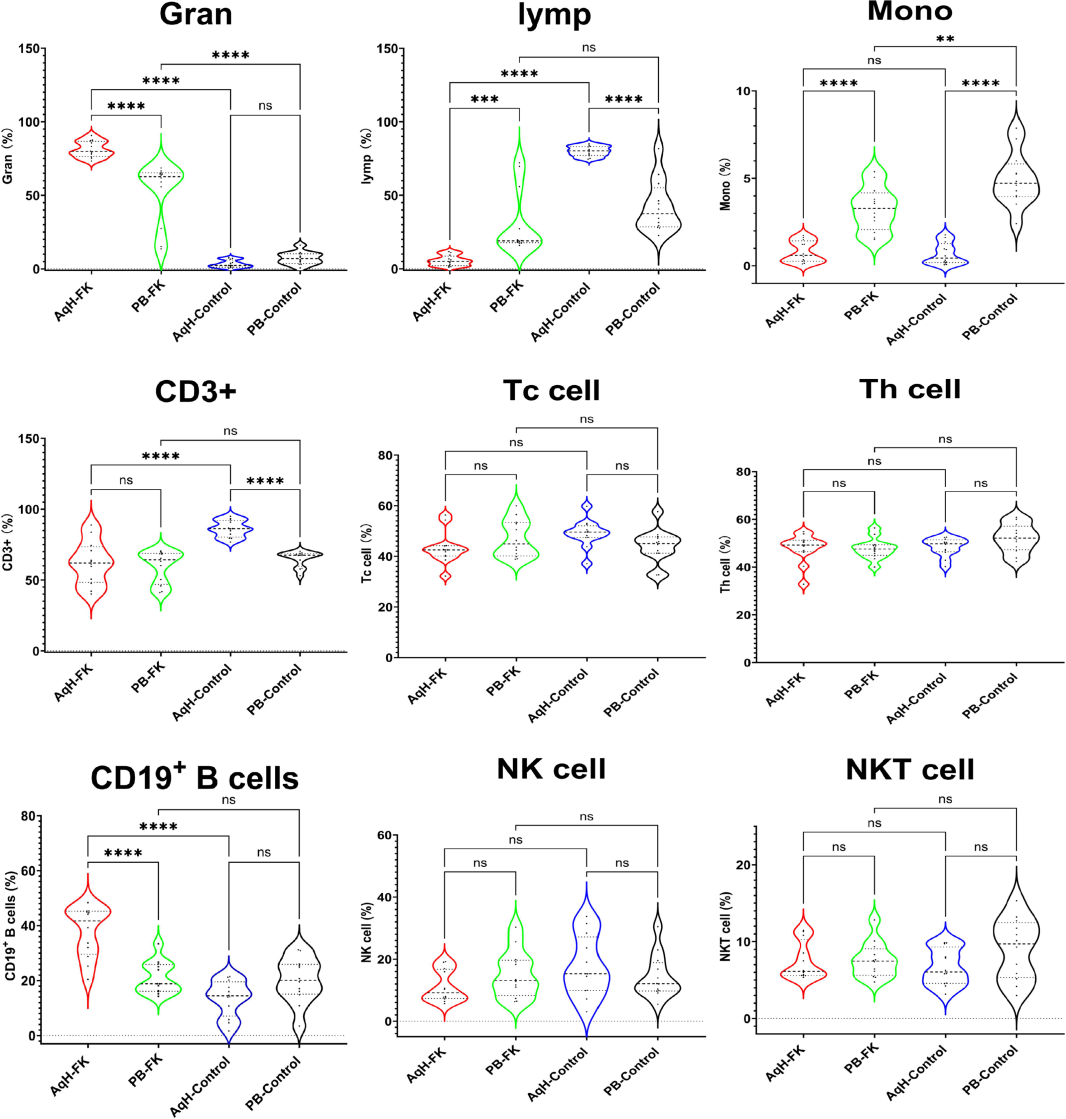
Cellular components in AqH and PB from FK patients and control. * indicates p < 0.05, ** indicates p < 0.01, *** indicates p < 0.001, and **** indicates p < 0.0001, while 'ns' denotes 'not significant' (p ≥ 0.05).

Further analysis of the lymphocyte compartment in aqueous humor revealed that FK patients exhibited a significantly higher proportion of B cells (approximately 30.70% of total lymphocytes) compared with controls, whereas the proportion of CD3+ T cells was relatively decreased. Additionally, the absolute lymphocyte count in aqueous humor was significantly lower in FK patients than in controls. No significant differences were observed in other lymphocyte subpopulations (**Table 2**; **Figure 3**).

**Table 2:** cellular components in AqH and PB from FK patients and control.

	Cellular components (%)				P-value		
Test Parameter	AqH-FK N=12	PB-FK N=12	AqH-control N=12	PB-control N=12	AqH-FK *vs*. PB-FK	AqH-FK *vs*. AqH-control	PB-FK *vs*. PB-control
Gran	81.25 (73.31-90.65)	72.17 (63.59-78.59)	3.32 (0.40-7.47)	71.26 (70.60-86.13)	<0.0001*	<0.0001*	0.7586
Lymp	5.56 (1.13-11.64)	23.86 (15.89-42.09)	82.01 (74.57-85.12)	25.40 (22.63-41.70)	0.0010*	<0.0001*	0.2627
Lymp#	154 ± 13	1568 ± 258	1247 ± 54	1657 ± 179	0.6872	<0.0001*	0.3687
Mono	0.81 (0.11-1.74)	3.26 (1.50-5.38)	0.69 (0.06-1.77)	4.94 (2.41-7.87)	0.0782	0.9984	0.0732
T/lymp	60.19 (40.13-88.83)	52.02 (11.20-70.52)	76.18 (66.89-85.03)	60.84 (8.23-70.30)	0.9910	0.0100*	0.9990
Tc/T	40.82 (14.17-64.33)	47.05 (38.90-59.97)	51.59 (37.11-73.81)	47.94 (32.64-57.66)	0.2840	0.0647	0.6024
Th/T	51.23 (32.85-76.25)	52.76 (40.03-56.39)	48.41 (26.19-62.89)	52.05 (42.34-60.88)	0.9328	0.8987	0.9950
B/lymp	30.70 (20.44-48.37)	21.06 (14.26-33.39)	8.22 (1.71-21.29)	20.04 (3.38-31.18)	<0.0001*	<0.0001*	0.8366
NK/lymp	8.22 (5.67-19.19)	25.17 (6.27-30.29)	15.59 (3.04-23.76)	14.34 (5.45-30.47)	0.0899	0.0569	0.6916
NKT/lymp	14.15 (5.35-75.60)	7.61 (4.75-12.83)	7.57 (3.17-20.88)	9.05 (2.99-15.32)	0.1375	0.1973	0.9957

*Indicates that the result is statistically significant.Lymp#, absolute lymphocyte count (cells/μL).

### 3.4 Significantly elevated pro-inflammatory cytokine concentrations in the aqueous humor of FK patients

**Figure 4:**
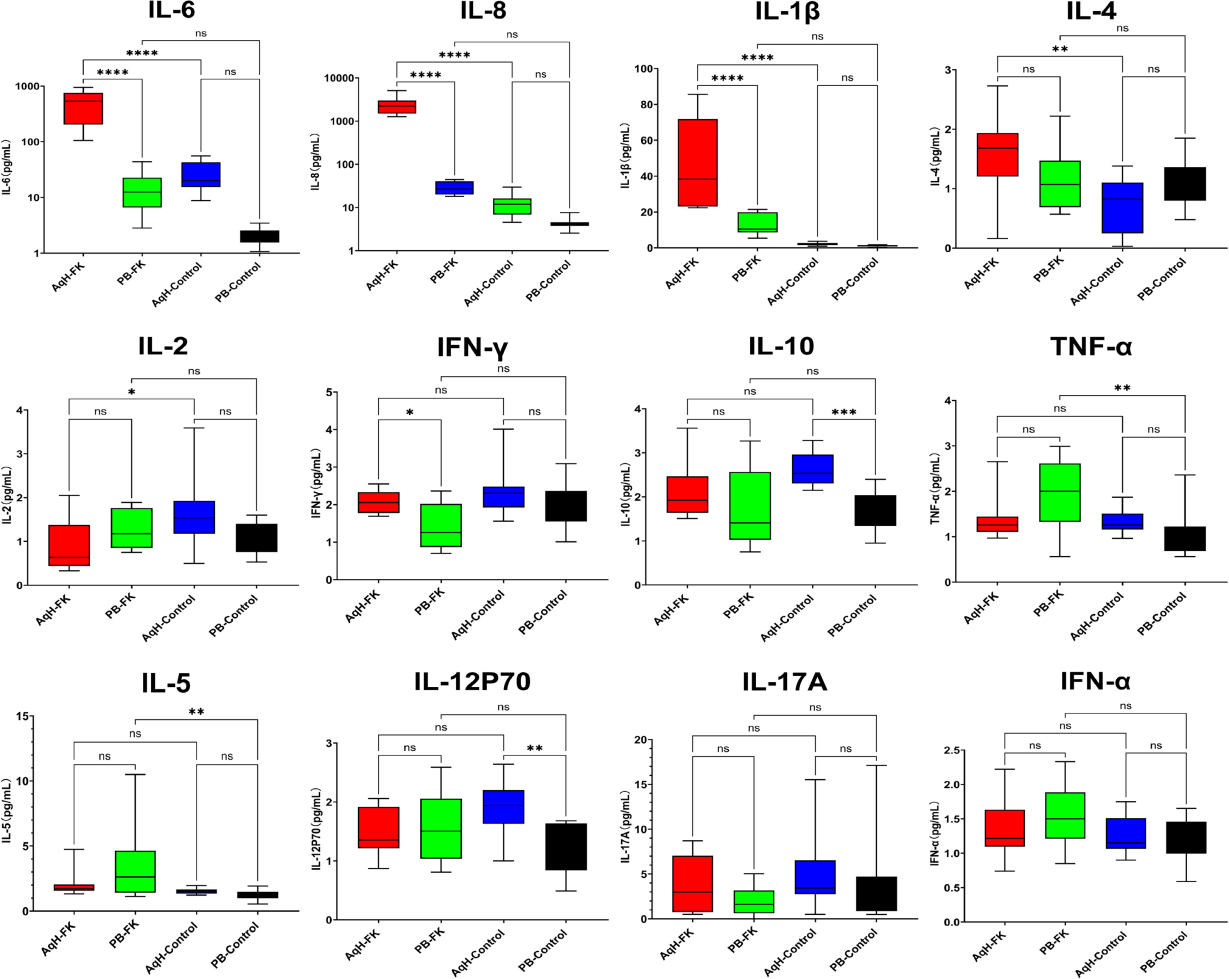
Concentrations of cytokines in AqH and PB from FK patients and control. * indicates p < 0.05, ** indicates p < 0.01, *** indicates p < 0.001, and **** indicates p < 0.0001, while 'ns' denotes 'not significant' (p ≥ 0.05).

Cytokine analysis revealed that the concentrations of IL-6 and IL-8 in the aqueous humor of FK patients were approximately 20-fold and 200-fold higher, respectively, compared with controls. Other pro-inflammatory cytokines, including IL-1β and IL-4, were also significantly elevated in FK patients. Notably, IL-2 levels in the aqueous humor were lower in FK patients than in controls. No significant differences were observed in IL-10 or IL-12p70 concentrations in the aqueous humor between the two groups; however, these cytokines demonstrated a notable increase in peripheral blood. Despite the marked cytokine alterations observed in the aqueous humor of FK patients, no significant differences in peripheral blood cytokine levels were detected between FK patients and controls (**Table 3**; **Figure 4**).

**Table 3:** Concentrations of cytokines in AqH and PB from FK patients and control.

	Concentration (pg/mL)				P-value		
Test Parameter	AqH-FK N=12	PB-FK N=12	AqH-control N=12	PB-control N=12	AqH-FK *vs*. PB-FK	AqH-FK *vs*. AqH-Control	PB-FK *vs*. PB-control
IL-1β	49.70 (22.37-116.58)	11.95 (4.28-21.47)	2.28 (0.99-4.03)	1.35 (0.17-3.11)	<0.0001*	<0.0001*	0.0253*
IL-6	505.71 (105.44-942.15)	19.10 (2.82-81.48)	156.34 (8.78-1590.83)	2.81 (1.07-7.05)	<0.0001*	<0.0001*	0.0345*
IL-8	2487.26 (1259.96-5059.23)	26.42 (15.48-44.20)	22.68 (4.54-89.67)	5.10 (2.24-27.50)	<0.0001*	<0.0001*	0.0397*
IL-5	2.00 (1.33-4.74)	3.34 (1.13-10.50)	1.52 (1.23-1.96)	1.37 (0.54-3.35)	0.0819	0.8919	0.0082*
IL-2	0.96 (0.33-2.05)	1.38 (0.18-2.51)	1.62 (0.50-3.59)	1.31 (0.21-2.66)	0.1622	0.038*	0.6327
IL-10	2.10 (1.51-3.56)	1.95 (0.70-5.11)	3.05 (2.15-8.28)	1.80 (0.50-3.30)	0.9652	0.0907	0.0769
IFN-α	1.34 (0.74-2.22)	1.45 (0.63-2.33)	1.25 (0.90-1.75)	1.45 (0.09-6.04)	0.9641	0.9924	0.8484
IL-12p70	3.47 (0.87-6.68)	1.66 (0.12-3.76)	4.89 (1.00-8.64)	1.25 (0.01-2.27)	0.8317	0.4127	0.737
IFN-γ	2.06 (1.69-2.55)	1.67 (0.22-5.69)	2.34 (1.56-4.01)	1.79 (0.64-3.09)	0.4968	0.8358	0.0808
IL-4	21.52 (0.16-82.73)	1.22 (0.51-2.38)	30.75 (0.03-91.38)	1.26 (0.48-2.76)	0.0668	0.0841	0.0532
TNF-α	4.44 (0.97-12.65)	3.14 (0.28-9.21)	5.32 (0.96-11.87)	4.36 (0.20-9.04)	0.1855	0.9922	0.0911
IL-17A	3.68 (0.50-8.71)	2.14 (0.28-7.21)	1.32 (0.96-1.87)	1.36 (0.20-3.04)	0.1855	0.9922	0.0911

*Indicates that the result is statistically significant.

Additionally, three hyperglycemic patients in the control group exhibited significantly higher aqueous humor levels of IL-4, IL-12p70, and TNF-α compared with normoglycemic controls. A similar cytokine profile was observed in two hyperglycemic patients within the FK group. Following exclusion of hyperglycemic patients from the analysis, no substantial differences in IL-4, IL-12p70, or TNF-α concentrations were observed between the FK and control groups.

### 3.5 Diminished adaptive immune cell function and increased immunosuppressive cell populations in the ocular microenvironment of FK patients

Analysis of T cell function revealed that FK patients had significantly lower proportions of activated T cells, CD28+ Th cells, and activated Tc cells in their aqueous humor compared with both their peripheral blood and the aqueous humor of controls. Within the B cell compartment, memory B cells were significantly reduced, whereas naïve B cells were significantly increased in the aqueous humor of FK patients compared with both peripheral blood and controls.

Compared with peripheral blood and controls, FK patients demonstrated a significant increase in Breg cells in their aqueous humor, accompanied by a higher proportion of Treg cells exhibiting an activated phenotype. The proportion of MDSCs was also elevated, with PMN-MDSCs representing the predominant subtype.

**Figure 5:**
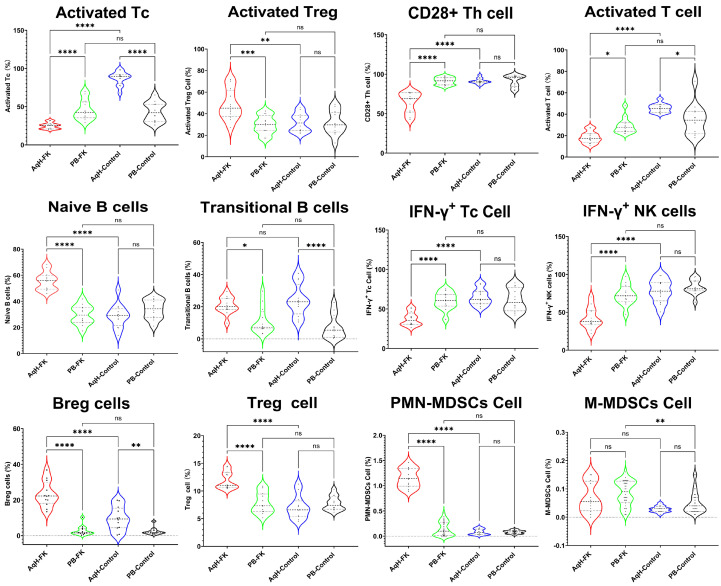
Immune cell functions components in AqH and PB from FK patients and control. * indicates p < 0.05, ** indicates p < 0.01, *** indicates p < 0.001, and **** indicates p < 0.0001, while 'ns' denotes 'not significant' (p ≥ 0.05).

With respect to cellular immune function, the capacity of NK cells and Tc cells to secrete IFN-γ was significantly diminished in the aqueous humor of FK patients compared with both peripheral blood and controls (**Table 4**; **Figure 5**).

**Table 4:** Immune cell functions components in AqH and PB from FK patients and control.

	Cellular components (%)				P-value		
Test Parameter	AqH-FK N=12	PB-FK N=12	AqH-Control N=12	PB-control N=12	AqH-FK *vs*. PB-FK	AqH-FK *vs*. AqH-control	PB-FK *vs*. PB-control
CD28+ Th/Th	70.90 (21.60-99.04)	91.17 (83.68-98.27)	87.85 (55.79-98.94)	89.85 (76.34-98.92)	<0.0001*	<0.0001*	0.9755
Activated T/T	25.10 (7.11-65.79)	35.98 (18.02-62.27)	47.80 (30.11-74.27)	30.35 (7.11-70.87)	<0.0001*	<0.0001*	0.9973
Activated Tc/Tc	30.24 (8.62-65.83)	41.58 (19.90-66.72)	87.20 (68.02-98.13)	40.15 (8.62-65.32)	<0.0001*	<0.0001*	0.7477
Activated Treg/Treg	48.98 (27.32-71.73)	30.35 (17.44-42.48)	32.73 (21.45-45.65)	30.91 (12.81-48.79)	<0.0001*	0.0006*	0.6644
Naive Th/Th	38.70 (23.99-50.79)	46.89 (28.17-66.10)	51.25 (24.04-74.02)	51.07 (22.85-72.19)	0.5733	0.2707	0.8001
Memory B/B	5.55 (3.67-18.29)	18.33 (2.77-39.21)	19.68 (3.29-24.53)	15.68 (4.48-28.27)	0.0089*	0.0002*	0.9999
Naive B/B	55.43 (49.33-68.21)	29.61 (17.26-41.80)	28.87 (13.96-48.71)	33.83 (21.04-43.63)	<0.0001*	<0.0001*	0.5226
Transitional B/B	25.04 (9.81-59.46)	11.21 (3.19-29.74)	24.05 (10.53-40.79)	7.16 (0.40-22.61)	0.0217*	0.8538	0.3355
PMN--MDSCs	1.30 (0.52-2.16)	0.23 (0.06-0.39)	0.02 (0.01-0.03)	0.09 (0.04-0.15)	<0.0001*	<0.0001*	0.1435
M--MDSCs	0.07 (0.00-0.15)	0.10 (0.03-0.16)	0.03 (0.01-0.05)	0.03 (0.01-0.08)	0.6281	0.0721	0.0042*
Treg/Th	11.19 (4.48-21.09)	7.52 (3.63-10.86)	6.80 (2.49-17.30)	7.91 (6.16-12.30)	<0.0001*	<0.0001*	0.965
Breg/B	23.21 (13.44-36.83)	2.81 (0.28-10.31)	9.61 (0.36-19.87)	2.38 (0.44-8.08)	<0.0001*	<0.0001*	0.9112
IFN-γ+ NK/NK	42.15 (21.76-70.22)	73.06 (47.41-97.27)	78.19 (40.45-98.69)	82.95 (63.46-97.06)	<0.0001*	<0.0001*	0.0055*
IFN-γ+ Tc/Tc	56.61 (26.49-80.27)	60.24 (36.98-75.39)	64.81 (50.98-82.46)	61.17 (42.25-83.04)	<0.0001*	<0.0001*	0.9805

*Indicates that the result is statistically significant.

### 3.6 Elevated blood glucose levels are associated with enhanced pro-inflammatory cytokine secretion and immunosuppression in the ocular microenvironment

**Figure 6:**
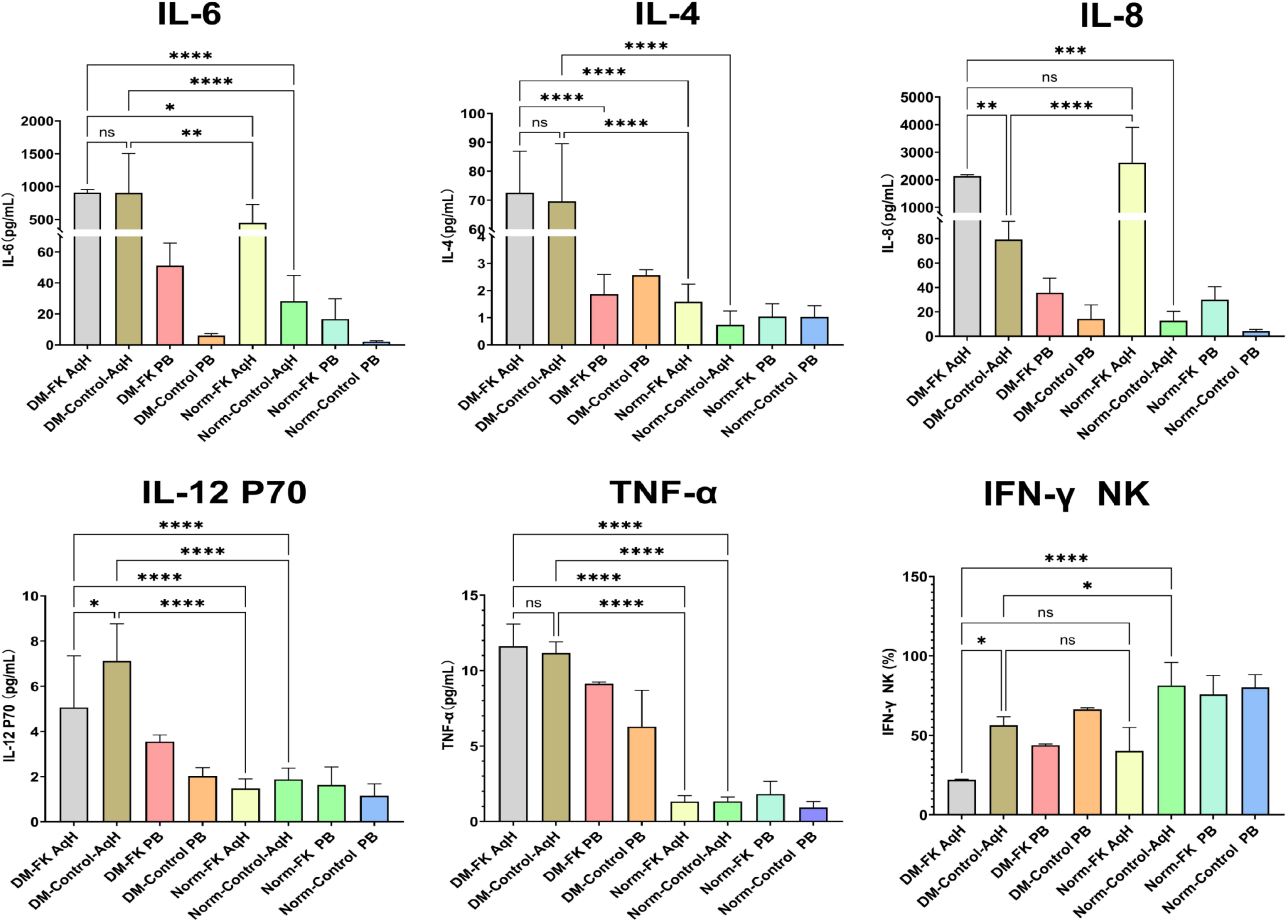
Comparison of cytokine levels and immune cell function in AqH and PB between the hyperglycemic group and normoglycemic group.

This study included 2 patients with poorly controlled type 2 diabetes mellitus in the FK group and 3 in the control group. Compared with normoglycemic patients within the same group, hyperglycemic patients exhibited significantly higher concentrations of IL-4, IL-6, IL-8, TNF-α, and IL-12p70 in their aqueous humor. Further analysis revealed that adaptive immune cells in the aqueous humor of hyperglycemic patients displayed a more pronounced immunosuppressive phenotype, and the capacity of NK cells to secrete IFN-γ was further diminished (**Figure 6**).

Given the limited sample size of the hyperglycemic subgroup (n = 2 FK patients; n = 3 controls), these findings should be regarded as preliminary and exploratory. The data suggest that hyperglycemia may be associated with elevated pro-inflammatory cytokine concentrations in the aqueous humor and an enhanced immunosuppressive phenotype; however, validation in larger, dedicated cohorts is required before definitive conclusions can be drawn.

## 4 Discussion

Fungal keratitis (FK) is a severe corneal infection that imposes a substantial global health burden, often resulting in vision loss if not promptly and effectively treated. Our epidemiological analysis of 145 FK patients in this region between 2018 and 2024 revealed a strong association between FK incidence and agricultural activities, with a predominance of middle-aged and elderly males (male-to-female ratio: 1.9:1) and corneal trauma from agricultural injuries as the primary causative factor (73.10%). Fusarium species were identified as the predominant pathogens (44.12%), consistent with findings from other tropical and subtropical regions (Carvalho, Cintra et al., 2024). The peak incidence occurred in October and November, coinciding with the local apple harvest season, further supporting the link between occupational exposure and FK. These epidemiological findings provide robust insights into the regional characteristics of FK and may inform public health interventions and clinical management strategies.

Beyond the epidemiological analysis, this study sought to characterize the immune microenvironment in the aqueous humor (AqH) of FK patients, which is essential for understanding disease progression and identifying potential therapeutic targets. Analysis of AqH revealed a distinctive immune profile in FK patients. Neutrophils were the predominant infiltrating cells (approximately 81.25%), significantly exceeding levels observed in the control group. This neutrophil dominance is characteristic of acute infection, reflecting a robust innate immune response against fungal pathogens (Mills et al., 2020; Abbondante et al., 2023; Vikelouda et al., 2023). In contrast to the control group, where lymphocytes constituted the most common infiltrating population, the proportion of lymphocytes in the AqH of FK patients was significantly reduced. Further analysis revealed that the proportion of B cells (approximately 30.70%) was significantly higher in the AqH of FK patients compared to controls, whereas the proportions of T cells and NK cells were relatively diminished. These findings suggest a complex interplay between innate and adaptive immune components within the FK ocular microenvironment, characterized by an enhanced innate immune response coexisting with altered adaptive immune function.

Regarding adaptive immune cell function, we observed that the proportions of activated T cells, CD28+ Th cells, and activated Tc cells in the AqH of FK patients were all significantly lower than in their peripheral blood and the AqH of the control group. Although the proportion of total B cells was elevated in the AqH of FK patients, this population consisted predominantly of naive B cells, with a significantly reduced proportion of memory B cells—cells critical for antigen-specific immune responses. Furthermore, the capacity of NK cells and Tc cells to secrete IFN-γ was significantly diminished. Collectively, these data suggest impaired adaptive immune function within the ocular microenvironment of FK patients.

We also observed an increased proportion of immunosuppressive cell populations in the AqH of FK patients, including Breg cells, activated Treg cells, and PMN-MDSCs. These cell types are known to modulate immune responses and maintain immune tolerance, typically by suppressing T cell and NK cell activity and producing anti-inflammatory cytokines (Ostrand-Rosenberg et al., 2023; Lu et al., 2024). The expansion of these immunosuppressive populations, coupled with reduced adaptive immune function, suggests an ocular microenvironment that may favor fungal persistence and chronic inflammation rather than effective pathogen clearance. However, it must be emphasized that these observations are correlative; the specific mechanisms by which these cells contribute to FK pathogenesis require further investigation through larger cohort and functional studies (Peng et al., 2020; Yu et al., 2023).

Cytokine profiling of AqH further corroborated these immunological alterations. We observed significantly elevated levels of pro-inflammatory cytokines in FK patients compared to controls, including IL-1β, IL-6 (approximately 20-fold elevation), and IL-8 (approximately 200-fold elevation). Elevated IL-1β can induce the production of IL-6 and IL-8, establishing positive feedback loops that perpetuate inflammation (Zhu et al., 2020; Wang and Tao, 2021; Duran-Ospina et al., 2024). Previous studies indicate that prolonged elevation of IL-6 and IL-8 can promote T cell exhaustion and impair immune regulation (Han et al., 2020; Ji et al., 2020; Peng et al., 2020). Elevated IL-8 can also recruit myeloid-derived suppressor cells (MDSCs) and inhibit the cytotoxic activity of natural killer (NK) cells (Abbondante et al., 2023; Fu et al., 2025), findings consistent with our observations of increased MDSCs and reduced NK cell function. These cytokine alterations, particularly the marked elevation of IL-6, suggest a central role for this cytokine in shaping the inflammatory and potentially immunosuppressive ocular microenvironment in FK. While IL-6 is critical for host defense, its dysregulation and persistent elevation may contribute to immunopathological damage. Therefore, early administration of IL-6-targeting agents (e.g., tocilizumab) could represent a potential strategy to alleviate ocular inflammation and reduce complications in FK (Peng et al., 2020). However, given the essential role of IL-6 in antifungal host defense, its blockade might compromise pathogen clearance (Peng et al., 2020). As a speculative hypothesis, this therapeutic approach requires rigorous preclinical research and clinical trials to evaluate its safety and efficacy prior to clinical implementation.

Our preliminary analysis of hyperglycemic patients suggested that elevated blood glucose levels may be associated with increased concentrations of IL-4, IL-6, IL-8, TNF-α, and IL-12p70 in the aqueous humor, accompanied by a more pronounced suppressive phenotype in adaptive immune cells and further diminished NK cell IFN-γ secretion (Yu et al., 2023). These immunological findings may reflect the immune status of patients with severe or late-stage FK, or those refractory to conventional treatment. The immune profile of patients with early-stage FK or those responding favorably to treatment may differ substantially. This clinical context is crucial for interpreting and generalizing our findings.

Finally, we acknowledge that the immunological and cytokine analyses were performed on a limited number of aqueous humor samples (n = 12 FK patients; n = 12 controls). This small sample size inherently limits statistical power and generalizability (Peng et al., 2020); accordingly, these immunological findings should be interpreted as exploratory observations, and larger, dedicated cohort studies are required to draw definitive conclusions. Additionally, the control group in this study comprised glaucoma patients undergoing concurrent surgery, selected to provide a source of comparable intraocular fluid samples (Yu et al., 2023). We acknowledge that glaucoma itself may be associated with a low-grade inflammatory response, which could potentially influence baseline intraocular cytokine levels (Wang and Tao, 2021; Yu et al., 2023). However, given the significant ethical and practical constraints inherent in obtaining aqueous humor samples from healthy individuals, the use of glaucoma patients as controls remains a methodologically reasonable approach (Xiao et al., 2022; Duran-Ospina et al., 2024). Future studies would benefit from including more immunologically quiescent control populations, if ethically and practically feasible.

## 5 Conclusion

Fungal keratitis (FK) is a severe ocular infection that can cause significant visual impairment or irreversible blindness. The epidemiological analysis of 145 FK patients demonstrated a strong association with agricultural activities in this region, with Fusarium species as the predominant causative pathogen. Despite the limited aqueous humor sample size (n = 12 per group), immunological profiling revealed notable alterations in the intraocular microenvironment, including neutrophil predominance, diminished T cell and NK cell function, B cell expansion, and increased immunosuppressive populations (Breg cells, activated Treg cells, and PMN-MDSCs). These findings suggest an immune milieu potentially conducive to chronic fungal infection and identify IL-6 as a candidate therapeutic target. However, the immunological observations should be interpreted as hypothesis-generating given the small sample size and potential confounding inherent to the glaucoma control group. Validation in larger, prospective cohorts is required before the therapeutic significance of these findings can be established.

## Data Availability

The raw data supporting the conclusions of this article will be made available by the authors, without undue reservation.
